# Typology of Health-Related Behavior: Hierarchical Cluster Analysis Among University Students

**DOI:** 10.3390/bs15070918

**Published:** 2025-07-07

**Authors:** Joca Zurc, Matej Majerič

**Affiliations:** 1 Department of Pedagogy, Faculty of Arts, University of Maribor, 2000 Maribor, Slovenia; 2 Faculty of Sport, University of Ljubljana, 1000 Ljubljana, Slovenia; matej.majeric@fsp.uni-lj.si

**Keywords:** higher education, student health, healthy lifestyle, well-being, cluster analysis

## Abstract

Physical and mental health show strong associations with health-related behavior. University students are one of the at-risk groups who are in a vulnerable transition phase from adolescence to adulthood, significantly affecting their health-related lifestyle. This study aims to identify different groups of university students with homogeneous health-related behavior, considering their dietary habits, physical activity, alcohol and tobacco consumption, mental well-being and lifestyle change motives. For data collection, an anonymous, closed-ended paper-and-pencil questionnaire was administered to a sample of 171 university students. Data analysis was performed using descriptive statistics, a *t*-test for independent samples, a chi-square test, Spearman correlation and hierarchical cluster analyses (Ward’s method, Dendrogram). On average, students reported good health (M = 4.84), including daily physical activity (M = 31.35 min) and regular consumption of fruits (M = 4.02) and vegetables (M = 4.19). The hierarchical cluster analysis revealed two distinct patterns among the students: “Caring for a healthy lifestyle” (N = 69) and “Physically inactive with poor mental well-being” (N = 62). Better health-related behavior was found among male students enrolled in higher study years (*p* ≤ 0.01). These findings provide new insights into the different patterns of health-related behavior among university students that require targeted health promotion actions. Universities should develop and implement courses in healthy lifestyles and sustain them in the curricula.

## 1. Introduction

Lifestyle is determined by a set of distinct health-related behaviors over a certain period of time, which could contribute to a healthy or unhealthy way of life. It is formed in childhood, under the influence of experiences and living conditions, and is influenced by the behavior of parents and immediate family circumstances, as well as other environmental, social, economic and cultural factors (Maučec Zakotnik, 2012). Recently, due to the established links between the occurrence of chronic non-communicable diseases (cardiovascular diseases, cancer, diabetes, etc.) and unhealthy lifestyles, interest in research in this area has increased both in Slovenia (Škof, 2010) and internationally (Emerole et al., 2025; Sakaria & Indongo, 2025).

Young people are a particularly high-risk group, as it has been found that health-related behavioral patterns that young people adopt at an early stage are carried over into adulthood and are significantly associated with cardiovascular prevention (Aira et al., 2023; Lawrence et al., 2017). Different influential factors contribute to healthy lifestyle behavior in university students. For example, cultural–social patterns and social relationships were found to have an important impact on lifestyle and health behavior among university students in the Czech Republic and Ukraine (Hrubá & Tanklevska, 2019). Further, statistically significant associations between the students’ socio-demographic characteristics and health-related behavioral risk factors were found among Nigerian students (Emerole et al., 2025). At the same time, the student’s grade, the educational level of their parents, the economic status of the family, and the place of student’s residence resulted in a significant difference in healthy lifestyle behavior in Turkish university students (Hacihasanoglu et al., 2011). Further, a student’s sports attitude was found to be a moderately positive influential determinator of their healthy lifestyle behavior. It seems that university students are aware of sports as an essential factor in stress management, coping with depression, physical ability, regular eating habits, and contributing to overall health (Yalcin et al., 2020). On the other hand, a healthier diet and non-smoking were found to be associated with more favorable physical activity development in young adults (Aira et al., 2023).

Based on WHO (2024) recommendations, a healthy lifestyle includes health-related behavior, such as eating a healthy diet with a combination of different foods, including lots of fresh fruits and vegetables, reducing harmful fats, sugar and salt intake; avoiding smoking and breathing tobacco-smoke-free air; exercising and maintaining a healthy body mass index (BMI). Following these recommendations and the study’s findings, different areas of lifestyle and social-demographic factors are strongly related. Therefore, they must be studied together simultaneously to embrace their interactions, which brings a new quality to the health-related behavior of an individual.

### Healthy Lifestyles of Slovenian University Students

Slovenia has almost 25 years of experience of measuring health-related behavior of adults in international comparable studies based on the methodology of non-communicable disease prevention. However, the population of university students or young adults between 18 and 24 years of age was studied for the first time in a cross-sectional survey in 2020 (Pustivšek et al., 2023), which means that this group of population was in the past not very much considered a risk of their health-related lifestyle, and as such, did not receive much attention, monitoring and evaluation from the national health and school politics. However, studies on students’ healthy lifestyles have become more popular since the beginning of the 21st century.

According to Bajt and Jeriček Klanšček (2014), young Slovenian people’s awareness of the importance of a healthy lifestyle for their health is high, as they critically recognize stress loads (91.5%), smoking (85.7%), obesity (84.1%), unhealthy diet (83.8%), alcohol consumption (83.6%) and lack of exercise (83.0%) as the strongest health-related risk factors. However, most (87.9%) evaluate their health as good, including 35.9% as excellent, but one-third of the Slovenian student population think that they do not care for their health well (Pustivšek et al., 2023).

Approximately 45.1% of Slovenian young people experience stress occasionally, and 25.3% often (Bajt & Jeriček Klanšček, 2014). Among the Slovenian adult population, the 18–24 years age group experiences stress more often than any other age group until 74 years. Almost 40% of young females defined themselves as experiencing stress, pressure or feeling tense daily. More than 4% of them coped with stress with difficulty and lack of control (Pustivšek et al., 2023). The proportion of adolescents who are at risk of experiencing feelings of depression has increased by around 10% in recent years (Jeriček Klanšček et al., 2023). Particularly, more stress is experienced under the pressure of schoolwork (Jeriček Klanšček et al., 2018). The end of the academic year is one of the most stressful periods for students, as they have a lot of work to do, submitting written assignments and studying for exams. Many experience personal defeat during this period if they perceive that they have not achieved their goals (Podjed, 2015). All these stress triggers increase the risk of health-related behaviors, such as the consumption of larger amounts of alcohol and tobacco, unhealthy eating and a lower amount of physical activity (Bajt & Jeriček Klanšček, 2014). Interestingly, according to a study by Podjed (2015), female and male students react differently to stress. Although male students report more stressful events than female students, they evaluate them more positively than female students. Jeriček Klanšček et al. (2018), in a Slovenian national study on the mental health of children and adolescents, found that stress is manifested in increasing psychosomatic symptoms. A total of 27.9% of adolescents reported experiencing at least two psychosomatic symptoms per week, such as insomnia, nervousness, irritability, and depression, increasing with age and female gender.

In Slovenia, 3600 people a year die from diseases related to tobacco smoking. On average, adolescents smoke less per day than adults, but most start smoking in their teens and early twenties, which can lead to smoking in adulthood. It has been found that individuals who start smoking at a younger age progress more quickly to becoming regular smokers, have more difficulty quitting smoking, and are also more susceptible to smoking-related diseases later in life (Zorko & Koprivnikar, 2015). In Slovenia, 23.5% of 15-year-olds have already smoked tobacco. Of the proportion of 15-year-olds who have already smoked tobacco, 9.6% of teenagers smoke at least once a week. The trend of tobacco smoking among adolescents has been decreasing in the last twenty years. However, electronic cigarettes are becoming more problematic, with 34.4% of 17-year-olds having already tried them and 10.5% smoking them regularly (Jeriček Klanšček et al., 2023).

Studies showed that the eating habits of Slovenian students mostly do not meet the characteristics of healthy eating (Dervišević & Vidmar, 2011; Pustivšek et al., 2023). Despite being aware of the importance of a healthy diet, students too often skip breakfast, have too few daily meals, and consume too few vegetables, fruit, and fish (Zupančič & Hoyer, 2006). The national study, which included 127 students from public faculties in Slovenia, found that students or young adults are the least likely to follow advice on healthy eating. They often skip meals; indeed, 14.2% of students never eat breakfast, more than half of students eat only two or fewer meals a day and only 34.6% of students eat all three main meals (breakfast, lunch and dinner) every day. Regarding food choices, students and young adults have a predominantly unhealthy diet, consuming too much sugar, salt and fat, and too little fruit, and especially vegetables, on average. Their diet is dominated by cereals and cereal products, starchy foods, meat products (fresh cured and dried sausages, salami and poultry), bananas, dairy products and dairy desserts (puddings), sweet drinks and multivitamin drinks and sweets (chocolate, candies, cakes and sweet and savory pastries) (Gabrijelčič Blenkuš, 2009). Recent results showed that the eating habits of the Slovenian population in the last 25 years have slightly improved, especially in terms of regularly eating breakfast and decreasing the consumption of sweet drinks. However, in eating breakfast and vegetables, 18–24-year-old Slovenians are still the lowest among the Slovenian adult population. Less than 50% of men and 60% of women in this age group eat breakfast daily, and only 24.2% of men and 35.4% of women in this age group eat vegetables daily (Pustivšek et al., 2023).

Alcohol consumption among young people in Slovenia is problematic. A total of 15.2% of adolescents drink alcohol regularly, 2–3 times a week, 43.7% drink alcohol 2–4 times a month, and 37.9% drink alcohol once a month (Hovnik Keršmanc et al., 2015). According to Lovrečič & Lovrečič (2014), the percentage of excessive alcohol drinkers among young people has been stable since 2001, and it is around 9–10%.

Sports and physical activities are university students’ most popular extracurricular activities (Djomba, 2014; Majerič, 2015). In the last forty years, the active student population in sports increased from 56.0% (Petkovšek, 1980) to 77.9% (Majerič, 2015). Students value sports as an important part of their lives and exercise regularly, mostly in moderate- or low-intensity activities, such as walking, jogging, swimming and cycling (Djomba, 2014; Drev, 2010). However, with increasing age, the level of physical activity is decreasing, especially among the most active groups (Jeriček Klanšček et al., 2023). These studies showed a positive trend in the increase in the share of Slovenian students who are regularly involved in sports and have a healthy lifestyle. However, on the other hand, Slovenian university students are, along with the highly educated residents with intellectual and sedentary professions, among the most sedentary groups of the Slovenian adult population, with an average of 7.1 h of sitting per working day and 6.1 h of sitting per day on weekends (Pustivšek et al., 2023). A total of 32.8% of adolescents spend more than four hours a working day in a sedentary position, which increases significantly with age. The trend of excessive sitting behavior of young people has been particularly evident from 2008 and onwards (Jeriček Klanšček et al., 2023).

Based on the presented findings, different socio-demographic factors and areas of health-related behavior significantly interact and shape an individual’s health-related lifestyle among university students. Therefore, our study aimed to examine dietary habits, physical activity and sports participation, alcohol and tobacco consumption, mental well-being and willingness for lifestyle changes among students at the University of Ljubljana in Slovenia. We were interested in identifying groups of students with homogeneous or similar patterns of health-related behaviors that were clearly distinguished from other groups in measured behaviors of a healthy lifestyle.

## 2. Materials and Methods

This study is based on a non-experimental empirical quantitative survey. It was carried out as part of the project entitled “Lifestyles Characteristics of University Students”.

### 2.1. Sample

A total of 171 students from the University of Ljubljana who selected the elective course of Sports Education participated in this study. Of these, 122 (71.3%) were female students and 49 (28.7%) were male students. Depending on the field of study, 37.6% were Communication Studies students (14.0% Marketing Communication and Public Relations, 12.1% Media and Communication Studies, 11.5% Journalism), 35.6% were Sociology students (14.0% Management of Human Resources, Knowledge and Organizations, 10.8% Analytical Sociology, 10.8% Social Informatics) and 23.6% were Political Science students (8.9% Defence and Security Studies, 4.5% Political Theory, Global and Strategic Studies, 3.8% European Studies, 3.2% Comparative Public Policies and Administration, 3.2% International Relations) and 3.2% were on the Cultural Studies program. Thus, the survey was conducted among students in most Bachelor’s and Master’s degree programs. The average age of the respondents was 21.5 ± 2.0 years; the youngest participant was 19 years old, and the oldest was 33 years old. However, 160 or 93.6% of the participants were between 19.5 and 23.5 years of age. The remaining 6.4 % of participants were evenly distributed between 24 and 33 years of age. Therefore, the individual participants with longer student status did not impact the data analysis and the findings. All study participants had an active and full-time enrolled student status in a study program at the University of Ljubljana. The selected sample presents a normal distribution of the Slovenian student population.

### 2.2. Measurement Tools

The data for the study were collected using a structured questionnaire, which was compiled based on the results of previous studies (Majerič & Markelj, 2009; Majerič & Zurc, 2016). The questionnaire consisted of several thematic sections: demographic data of the respondent, assessment of health and health status, physical measurements, dietary habits, physical activity, extracurricular activities, mental well-being and readiness for lifestyle changes. Interval measurement scales (1–4, 1–5, 1–6, 1–7) were mainly used. To a lesser extent, numerical (minutes of physical activity/day, alcoholic beverages/week), ordinal (hours of physical activity/week) and nominal (smoking, alcohol consumption) variables were also used. For data analysis, some interval scales were reversed (e.g., dietary habits, mental well-being) to point in the same direction of better health-related behavioral style. Namely, a higher value on the scale means a better behavioral style. Some variables were combined into a common variable (e.g., organization of physical activity, smoking, number of alcoholic beverages per week). Cronbach’s alpha, with a value of 0.447 for health assessment, 0.032 for physical activity, 0.442 for dietary habits, 0.582 for mental well-being and 0.701 for the assessment of reasons for lifestyle changes, indicated the lower reliability of the measurement instrument used.

### 2.3. Data Collection Procedure and Ethical Considerations

Data was collected at the Faculty of Social Sciences, University of Ljubljana, during the workshop on healthy lifestyles, which was organized for students in the first semester of the current academic year. Students were surveyed by their peers, kinesiology students from the Faculty of Sport, University of Ljubljana, who were specially trained to conduct this research. The same group of students provided all measurements, e.g., implementing questionnaires and direct body composition measurements using the TANITA TC 602 scale (TANITA CORP of America). The TANITA scale measured fat proportion in the whole body and gave average BMI values according to age.

The study was conducted according to the guidelines and ethical principles for research involving human subjects of the Declaration of Helsinki. Considering the absence of sensitive ethical issues, the voluntary and anonymous nature of the survey and the project evaluation by peers at the Faculty of Sport, University of Ljubljana, assessment by another ethics committee was not required. However, all participating students provided written informed consent for their participation in the study before the data collection. All results are presented with respect for the confidentiality and anonymity of the participants.

### 2.4. Data Analysis

The data collected on the behavioral style of students in the field of health were analyzed using the SPSS 20.0 statistical package (SPSS Inc., Chicago, IL, USA) from the perspective of descriptive statistics (frequencies, percentages, mean values, standard deviation), bivariate analysis (*t*-test for independent samples, Spearman’s rank correlation coefficient, chi-square test) and multivariate cluster analysis. All inferential statistical tests were chosen based on the previous testing of collected data for the normal distribution using descriptive statistics (skewness, kurtosis) and the Kolmogorov–Smirnov test (*p* < 0.05).

Groups of students with homogeneous patterns of health-related behaviors were identified using the hierarchical clustering method (Ward’s method, Euclidean distance, dendrogram) (Ferligoj, 2011). Differences between the obtained groups according to selected socio-demographic characteristics (gender, age, BMI) were tested using the chi-square test and *t*-test for independent samples. Only results with a statistical significance level of 0.05 or less were considered statistically significant.

## 3. Results

The Results section provides the survey results, including descriptive, bivariate and multivariate statistics and their interpretation.

Table 1 shows that the surveyed students assessed their health as very good (M = 4.84) and similarly evaluated their self-knowledge and reactions in different health-related situations (M = 4.86). University students assessed their care for physical and mental well-being a little lower, but still had a high average score, of 4.11. Regarding dietary habits, regular or three-to-five-times-a-week consumption of vegetables (M = 4.19) and fruits (M = 4.02) stands out. Conversely, the surveyed students also consume fried food several times a week (M = 3.56) and sweets at least once weekly (M = 2.92). Among the evaluated dietary habits, students rated fish consumption the lowest, less than once a week (M = 2.29).

Physical activity is important in the lives of students (M = 4.42), being physically active two to three times a week (M = 4.99), for a total of three to five hours a week (M = 2.13) and more often in an unorganized form independently, e.g., with friends or family (M = 3.03). The average time spent performing daily physical activity, which is not performed in sports activities but increases the heart rate and breathing (e.g., cycling, walking to the university, daily household chores), is 31 min.

A total of 80.7% of students declared that they consume alcohol, drinking an average of 3.67 glasses per week. This includes a glass or can of beer (3 dcl), wine (2 dcl), aperitif (0.5 dcl), liqueur (0.5 dcl) or a mixture (3 dcl). Only 26% of students have never smoked. Smokers stated that they smoked mainly in the past, but today, they no longer smoke as often or do not smoke at all (M = 3.72).

In the area of mental health, the results show that students rated their sense of self-confidence the highest (M = 3.34), and they have not had any major problems with sleep (M = 3.06), attention (M = 2.98) or with feelings of constant pressure (M = 2.90) in the recent period. Their general satisfaction with life has increased (M = 2.68). However, the most important reason for changing their lifestyle among the surveyed students is an improvement in mental well-being (M = 4.94), followed by an improvement in physical well-being (M = 4.88) and the motive to be healthier (M = 4.73).

Bivariate inferential analysis showed statistically significant differences between male and female students in the care they devote to their physical and mental well-being (*p* = 0.001), frequency of eating fried food (*p* = 0.005), alcohol consumption (*p* = 0.019) and number of glasses of alcohol consumed per week (*p* = 0.019), the importance that students attribute to physical activity in their lives (*p* < 0.001), frequency (*p* < 0.001) and number of hours of physical activity per week (*p* < 0.001) and involvement in organized forms of physical activity (*p* = 0.011), where male students gave higher self-ratings in all lifestyle variables compared to female students. Female students were statistically significantly more likely to change their lifestyle due to better mental well-being (*p* = 0.002) and general health (*p* = 0.022) than male students.

With the age of students increasing, the frequency (*p* = 0.019), number of hours of physical activity per week (*p* = 0.033), and number of minutes of daily physical activity outside of sports activities that increase heart rate and breathing (*p* = 0.021) increase statistically significantly. With age, the frequency of eating fried food (*p* = 0.031) and sweets (*p* = 0.028) decreases statistically significantly. The value of the Spearman coefficient for the mentioned correlations ranges between 0.164 and 0.180 and indicates a weak connection between lifestyle variables and age.

BMI is a measure for indicating nutritional status in adults. It is defined as an individual’s weight in kilograms divided by the square of their height in meters (kg/m^2^). Normally nourished people have a BMI between 18.5 and 24.9 kg/m^2^. A BMI below 18.5 kg/m^2^ means underweight. Overweight or pre-obesity is indicated by a BMI between 25.0 and 29.9 kg/m^2^. A BMI greater than 30 kg/m^2^ indicates obesity, with a BMI between 30.0 and 34.9 kg/m^2^ indicating obesity class I, between 35.0 and 39.9 kg/m^2^ indicating obesity class II and above 40.0 kg/m^2^ indicating obesity class III (WHO, 2010). In our study, the average BMI for the entire sample of study participants (n = 171) was calculated to be 22.88 kg/m^2^, which indicates the normal nutritional status of the surveyed students. Despite the above, the lowest measured BMI, with a value of 17.40 kg/m^2^, also indicates the presence of malnourished students, and the highest measured BMI, with a value of 48.00, indicates the presence of obesity class III among students. The standard deviation of 3.77 kg/m^2^ indicates that the majority of the surveyed students were normally nourished with a BMI between 19.11 and 26.65 kg/m^2^. BMI is statistically significantly positively correlated with the number of hours of physical activity per week (*p* = 0.007) and involvement in organized physical activity (*p* = 0.039). However, both Spearman correlations were weak (*ρ* < 0.210).

**Figure 1:**
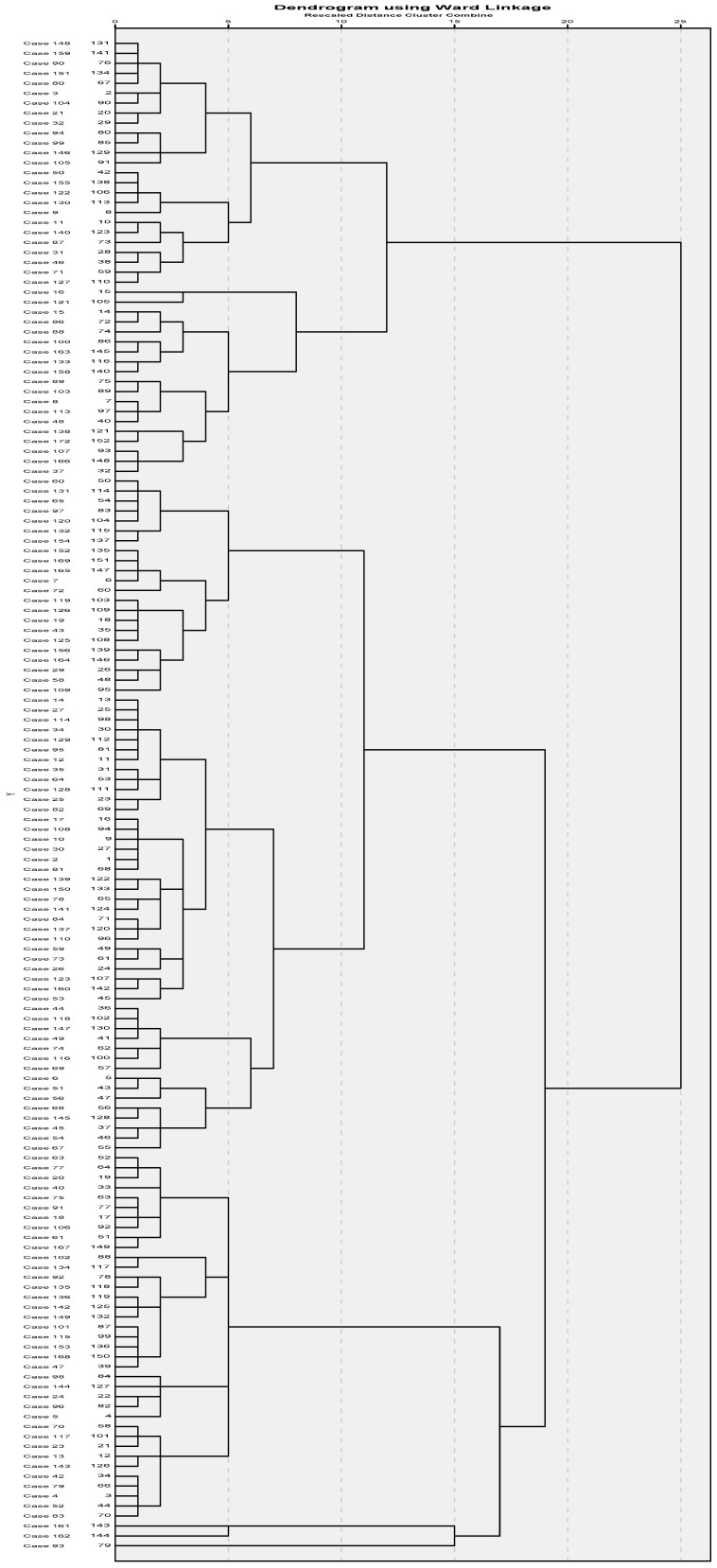
Dendrogram of hierarchical cluster analysis by Ward’s method.

The Ward’s hierarchical clustering dendrogram showed two distinct groups of students regarding health-related behaviors. Given the distances between the clustering levels or the measures of difference between the groups, we cut the tree at the first level, where the most significant jump between two adjacent clustering levels was evident from the graphical representation (Figure 1).

Of the 177 students surveyed, 40.4% were classified in the first group and 36.3% in the second group. 23.4% of students surveyed were not classified in the groups due to missing values for specific questions in the survey (Table 2). The obtained average values were evaluated for each of the studied variables in each group using a four-point scale according to the deviations from the average of the entire sample (Ferligoj, 2011):

- ++ deviations from the average higher than 0.30
- + deviations from the average higher than 0.15
− deviations from the average lower than 0.15
−− deviations from the average lower than 0.30

The sum of all deviations of the arithmetic means from the total average of the entire sample was also calculated for each group.

**Table 2:** Hierarchical cluster analysis (Ward’s method) with a two-group solution.

Characteristics	Total Sample (N = 171)	Group 1 (N = 69)	Group 2 (N = 62)	*t* (*p*) ^2^
	M (SD) ^1^	M (SD)	M (SD)	
Self-rated health ^3^	4.84 (0.81)	4.93 (0.83)	4.68 (0.76) − ^17^	1.791 (0.076)
Care for physical and mental well-being ^4^	4.11 (0.94)	4.47 (0.87) ++ ^15^	3.69 (0.84) −− ^18^	5.160 (<0.001)
Assessment of self-knowledge and response in different situations ^4^	4.86 (0.89)	5.10 (0.79) + ^16^	4.61 (0.91) −	3.263 (0.001)
Frequency of fruit consumption ^5^	4.02 (0.86)	4.33 (0.78) ++	3.77 (0.91) −	3.780 (<0.001)
Frequency of vegetable consumption ^5^	4.19 (0.95)	4.51 (0.70) ++	3.82 (1.08) −−	4.257 (<0.001)
Frequency of fish consumption ^5^	2.29 (0.85)	2.41 (0.96)	2.18 (0.69)	1.575 (0.118)
Frequency of fried food consumption ^6^	3.56 (0.92)	3.80 (0.88) +	3.42 (0.90)	2.425 (0.017)
Frequency of sweets consumption ^6^	2.92 (1.08)	2.97 (1.01)	2.84 (1.26)	0.666 (0.507)
Importance of physical activity in life ^7^	4.42 (1.23)	4.68 (1.17) +	3.84 (1.16) −−	4.129 (<0.001)
Frequency of physical activity ^8^	4.99 (1.02)	5.49 (0.83) ++	4.27 (0.83) −−	8.356 (<0.001)
Number of hours of physical activity per week ^9^	2.13 (0.92)	2.58 (0.81) ++	1.45 (0.53) −−	9.487 (<0.001)
Number of minutes of physical activity per day that increases heart rate and breathing	31.35 (22.84)	37.97 (26.81) ++	27.32 (17.77) −−	2.704 (0.008)
Organized physical activity ^10^	2.03 (0.72)	2.09 (0.75)	1.81 (0.48) −	2.574 (0.011)
Unorganized physical activity ^11^	3.03 (0.99)	3.24 (1.02) +	2.77 (0.92) −	2.761 (0.007)
Number of alcoholic beverages per week	3.67 (8.71)	4.51 (11.61) ++	1.98 (3.36) −−	1.727 (0.088)
Attention ^12^	2.98 (0.42)	2.99 (0.47)	3.02 (0.34)	−0.424 (0.672)
Difficulty sleeping due to worry ^13^	3.06 (0.95)	3.32 (0.85) +	2.90 (0.99) −	2.591 (0.011)
Feeling of constant pressure ^13^	2.90 (0.89)	3.06 (0.92) +	2.71 (0.89) −	2.190 (0.030)
Feeling of losing self-confidence ^13^	3.34 (0.86)	3.65 (0.59) ++	3.06 (0.96) −	4.178 (<0.001)
Satisfaction with life ^12^	2.32 (0.67)	2.49 (0.66) +	2.16 (0.63) −	−2.943 (0.004)
Reason for lifestyle change for better physical well-being ^7^	4.88 (1.27)	4.91 (1.37)	4.94 (1.14)	−0.101 (0.920)
Reason for lifestyle change for better mental well-being ^7^	4.94 (1.25)	4.75 (1.32) −	5.23 (1.02) +	−2.306 (0.023)
Reason for lifestyle change for better health ^7^	4.73 (1.48)	4.91 (1.39) +	4.82 (1.44)	0.365 (0.716)
Smoking ^14^	3.72 (1.48)	3.67 (1.46)	3.95 (1.46) +	−1.078 (0.283)
Sum of mean deviation in cluster based on total sample mean:		+11.55	−10.04	

Legend: ^1^ Mean value (M) and standard deviation (SD). ^2^ Coefficient and *p*-value of the statistical significance for *t*-test for independent samples. ^3^ Measured on a 6-point scale: 1 = least good to 6 = very good. ^4^ Measured on a 6-point scale: 1 = none to 6 = very good. ^5^ Measured on a 5-point scale: 1 = rarely or never to 5 = six or more times a week. ^6^ Measured on a 5-point scale: 1 = six or more times a week to 5 = rarely or never. ^7^ Measured on a 6-point scale: 1 = not important to 6 = very important. ^8^ Measured on a 7-point scale: 1 = never to 7 = every day. ^9^ Measured on a 4-point scale: 1 = zero or less than 3 h to 4 = more than 10 h. ^10^ Composite variable from six questions on different forms of organized exercise, which were assessed on a 6-point scale: 1 = very rarely or not at all to 6 = very often. ^11^ Composite variable from four questions on different forms of unorganized exercise, which were assessed on a 6-point scale: 1 = very rarely or not at all to 6 = very often. ^12^ Measured on a 4-point scale: 1 = much worse than usual to 4 = much better than usual. ^13^ Measured on a 4-point scale: 1 = much more than usual to 4 = much less than usual. ^14^ Measured on a 5-point scale: 1 = every day to 5 = never. ^15^ ++ = deviation from the sample average is higher than 0.30. ^16^ + = deviation from the sample average is higher than 0.15. ^17^ − = deviation from the sample average is lower than 0.15. ^18^ −− = deviation from the sample average is lower than 0.30.

The first group of students, compared to the average of the entire sample, are characterized by greater concern for their own physical and mental well-being (M = 4.47), more frequent consumption of vegetables (M = 4.51) and fruit (M = 4.33), greater frequency (M = 5.49) and number of hours of physical activity per week (M = 2.58), higher average time of daily movement outside of sports activities that increase heart rate and breathing (M = 38 min, 7 min more than in the entire sample), a greater number of glasses of alcohol consumed per week (M = 4.51) and a better sense of self-confidence (M = 3.65).

The second group represented students, who, compared to the average of the entire sample and the first group, were characterized by less concern for their own physical and mental well-being (M = 3.69), less frequent consumption of vegetables (M = 3.82), less importance of physical activity in life (M = 3.84), lower frequency (M = 4.27) and number of hours of physical activity per week (M = 1.45), lower average time of daily movement outside of sports activities that increase heart rate and breathing (M = 27 min, 4 min less than the entire sample), and a lower number of alcoholic beverages per week (M = 1.98, which is 1.69 alcohol drinks less than the average of the whole sample). The second group also had more non-smokers (M = 3.95) and expressed a stronger desire to change their lifestyle for better mental well-being (M = 5.23) (Table 3).

One-factor analysis of variance showed that the two obtained patterns of health-related behaviors statistically significantly differentiate students into two groups in caring for physical and mental well-being (*p* < 0.001), assessment of self-knowledge and response in different situations (*p* = 0.001), frequency of consumption of fruit (*p* < 0.001), vegetables (*p* < 0.001) and fried food (*p* = 0.017), attribution of the importance of physical activity in life (*p* < 0.001), frequency (*p* < 0.001) and a number of hours of physical activity per week (*p* < 0.001), average time of daily physical activity that is not performed in sports activities, but increases heart rate and breathing (e.g., bicycle transportation, walking to college, daily chores) (*p* = 0.008), and involvement in unorganized (*p* = 0.007) and organized physical activities (*p* = 0.011). In the area of mental health, the two groups differed statistically significantly in sleep problems due to worry (*p* = 0.011), feeling of constant pressure (*p* = 0.030), feeling of losing self-confidence (*p* < 0.001) and assessment of life satisfaction (*p* = 0.004). Statistically significant differences were also found between the two groups regarding the motive for changing lifestyles due to better mental health (*p* = 0.023). In all the differences found in the areas of self-assessment of health and health care, dietary and physical activity habits and mental health, the first group prevails in terms of a better lifestyle, except for the motive for changing lifestyle due to better mental well-being, which is stronger in the second group of students. Based on the findings obtained, we named the first group of students “Caring for a healthy lifestyle” and the second group “Physically inactive with poor mental well-being” (Table 3).

The analysis of socio-demographic differences between the two groups of students divided by their health-related behaviors showed statistically significant differences by gender (*p* < 0.001) and age (*p* = 0.009). The first group, “Caring for a healthy lifestyle”, is represented by mostly male students (84.4%), with an average age of 22 years. The second group, “Physically inactive with poor mental well-being”, has more female students (57.6%), with an average age of 21 years. The calculation of BMI did not show statistically significant differences between the two groups of students with different patterns of health-related behaviors.

## 4. Discussion

The summarized findings show that the results are consistent with similar studies in our country. In terms of self-assessment of health, we found a very good self-assessment, similar to Jeriček Klanšček et al. (2023), Klanjšek (2014) and Tomšič (2014). In terms of dietary habits, we found some similarities with the diet of the average Slovenian (Gabrijelčič Blenkuš & Kuhar, 2009; Hlastan Ribič & Kranjc, 2014b) and international populations (Gudelj Rakić et al., 2024). In our case, students do not eat enough fruits, vegetables, fish, and eat too often fried foods and sweets. The BMI analysis showed that most students are normally nourished, but among those surveyed, some were also malnourished and extremely obese. Given that only one-fifth of students were overnourished, these characteristics are more comparable to the nutritional status of children and adolescents up to the age of 19 (Grmek Košnik, 2011; Gudelj Rakić et al., 2024; Hlastan Ribič & Kranjc, 2014a) than adults (Fras & Leskošek, 2007). Similarly to Majerič and Markelj (2009), Majerič (2015) and Pustivšek et al. (2023), we found that physical activity is significant in the lives of students. Most of them are physically active in an unorganized form (independently, with friends or within the family) two to three times a week for a total of three to five hours a week. When analyzing smoking, it is interesting that only 26% of the surveyed students have never smoked. This figure is higher than the findings of Koprivnikar and Macur (2015). Smokers in our study identified themselves as having smoked mainly in the past, but today, they no longer smoke as much. They smoke frequently or not at all anymore. This suggests that the proportion of young smokers is decreasing, as was also found by Kirbiš (2014). The finding that 80.7% of students consume alcohol (drinking an average of 3.67 alcoholic beverages per week) is worrying. This proportion is higher than that shown in a comparable study (Hovnik Keršmanc et al., 2015). When assessing mental health and stress, we can summarize that approximately half of the surveyed students experience stressful situations which do not yet threaten their mental health. Bajt and Jeriček Klanšček (2014) also found a similar result. On the contrary, in the recent Slovenian survey (Pustivšek et al., 2023), almost 40% of young females between 18 and 24 years of age defined themselves as experiencing stress daily. When analyzing the reasons for changing their lifestyle, it is interesting that most respondents emphasized improving their mental well-being as the reason for changing, followed by improving their physical well-being and then being healthier.

In the analysis to identify groups of students with homogeneous patterns of health-related behaviors, we used the clustering method, which contributes to the recognition and understanding of different patterns of student lifestyles. We have not yet seen such an approach in the studied population in Slovenia. Ward’s hierarchical clustering method revealed two behavioral patterns of students related to their health: (1) students who take care of their healthy lifestyle and (2) students who have a sedentary lifestyle, which is also reflected in poorer mental well-being. The results obtained are comparable to some related research. A study on a sample of 1557 undergraduate nursing, midwifery and teaching students in Ireland also revealed two homogeneous groups of students’ health-related behaviors. Similarly to our research, in this study, the first group is represented by students with a positive behavioral style, and the second group is represented by students with risky behaviors, which mainly includes the health domain of poorer mental health, such as experiencing mental distress and their passive coping (Deasy et al., 2015). Using the clustering method, Doron et al. (2015) revealed four homogeneous groups of mental well-being management in a sample of 578 undergraduate students in France in medicine, dentistry, psychology and sports science. The group of students experiencing more stress and the group of students avoiding stress showed a worse health-related behavioral style compared to those adapting to stress and those experiencing less stress. The authors emphasized that the clustering method allows for a better distinction of students’ behavioral patterns in mental health and coping with stress compared to traditional statistical analyses. In our previous studies with clustering analysis on different populations (e.g., elderly, primary school children), we found similar results in terms of clear and systematic differentiations between more homogenous groups based on health-related behaviors (Zurc et al., 2015; Zurc & Laaksonen, 2023). Schneider et al. (2009) pointed out that the clustering method enables a complex study of several different behavioral habits, thus identifying characteristic health-related behavioral patterns. This makes it possible to detect different risk groups and was also revealed in our study of a sample of university students. It seems that the second cluster group, “Physically inactive with poor mental well-being”, detected the risk of female students, particularly at the beginning of the study. According to Podjed (2015), these results are expected because male students tend to use strategies that are focused on solving the problem, including planning, active coping, and accepting stress, while female students, on the other hand, predominantly use the strategy of seeking social support, which means that they usually need the help of other people to solve their problems. Therefore, the population of female university students should be taken into consideration for an in-depth analysis of the factors that influenced their lower physical activity and mental well-being status. The deterioration of mental well-being among the Slovenian student population was shown particularly during the COVID-19 pandemic, which significantly impacted the lifestyle of Slovenian students across various health domains, particularly mental well-being (Zurc, 2024). Our findings showed that these negative consequences of the pandemic should be actively addressed through organized health promotion interventions, especially targeting the student population. Although the National Institute of Public Health of the Republic of Slovenia (2022) is developing and implementing numerous valuable health promotion and health education programs, there is a lack of activities oriented towards the student population. These actions should be prioritized in the future to improve mental well-being, physical activity, and overall healthy lifestyles among the vulnerable student population.

Meanwhile, the cluster group “caring for health” was presented predominantly in male students compared to female peers who were only one year older. Age analysis of different lifestyles of students during their bachelor’s, master’s or PhD study would be very beneficial for future research and practice for target-oriented health promotion and health education programs. Before the Bologna Declaration in 1999 (European Higher Education Area, 1999), every university study program in Slovenia had an obligatory course in physical education, at least two study years, two hours per week during both semesters, a total of 60 h per academic year. After implementing the Bologna process, this course was withdrawn from most Slovenian universities and never returned to study programs in the same form. Jamnik (2022) highlights the critical impact of the Bologna reform on students’ sports activities at the University of Ljubljana, which immediately fell from around 40% before to 12.9% after reform implementation. However, in our neighboring country, Croatia, it is still in the curriculum under the name of Physical and Health Education, and it is obligatory for all students for at least two years. Therefore, it seems justified to advocate for at least an elective course on health education/promotion for students, particularly in study programs that educate future professionals working with children and young people, such as education, psychology, sociology, and other humanities and social sciences.

Our findings are important for practice, as we primarily determined the effectiveness of the clustering method in relation to health-related behaviors. The statistical approach proved to be useful and will enable us to more easily determine the characteristics of health-related behaviors in different population groups in the future. We are also aware of the limitations of this pilot study, which was conducted on a smaller sample of respondents. We would also like to highlight the lower reliability of the questionnaire used, especially in the questions on behavioral style in physical activity. For future surveys, we will refine these questions, as well as sets of questions on dietary habits, the self-assessment of health and mental health, and make them more approachable, understandable and convenient for the target respondents. For future studies on students’ lifestyle, it will also be crucial to measure some new forms of health-related risk behaviors, such as different substances of smoking (e.g., electronic cigarettes, tablets and other new drug substances) and alcohol consumption (e.g., mixed drinks). Similarly, also in the area of diet, it would be interesting to study different specific diets and lifestyle patterns among the youth population.

## 5. Conclusions

Monitoring health-related behavior is essential if we want to plan targeted health promotion-related measures and evaluate the effectiveness of their implementation. It serves politicians and professionals to prioritize their strategies and programs towards the most prominent health-related behavior issues and support the groups of the population that need support the most with their effective measures. The burden of an unhealthy lifestyle greatly threatens the entire population of Slovenia; it requires universal public health measures and the development of supportive environments that enable healthy choices for everyone, while the excessive burden of more vulnerable population groups with unhealthy behavior, such as university students, requires planned additional and specific measures to protect their vulnerability and more effectively strengthen their health (Tomšič, 2014).

By analyzing the data, we achieved the purpose of the paper, which was to study dietary and physical activity habits, alcohol and tobacco consumption, mental well-being and readiness for lifestyle changes among university students at the University of Ljubljana. The study results largely indicate the comparability of available studies conducted in our country in the recent two decades and support further national longitudinal monitoring of health-related behavior among university students. Despite fewer evident health-related issues within this vulnerable group in the transitional phase from adolescence to adulthood, this should not be neglected in targeted interventions to improve different aspects of their health-related behavior.

## Figures and Tables

**Table 1:** Descriptive statistics of students’ lifestyle distribution related to self-assessed physical activity, nutrition habits, mental health, alcohol and tobacco consumption, and testing statistically significant differences in gender, age and ITM.

Characteristics	(N = 171) M (SD) ^1^	Gender *t* (*p*) ^2^	Age *ρ* (*p*) ^3^	BMI ^4^ *ρ* (*p*)
Self-rated health ^5^	4.84 (0.81)	0.770 (0.442)	−0.014 (0.859)	−0.065 (0.402)
Care for physical and mental well-being ^6^	4.11 (0.94)	3.347 (0.001)	0.034 (0.662)	0.081 (0.297)
Self-knowledge and reactions in different health-related situations ^6^	4.86 (0.89)	1.655 (0.100)	−0.045 (0.560)	0.008 (0.917)
Frequency of fruit consumption ^7^	4.02 (0.86)	−1.408 (0.161)	0.026 (0.735)	−0.027 (0.726)
Frequency of vegetable consumption ^7^	4.19 (0.95)	−0.594 (0.553)	0.059 (0.443)	−0.031 (0.695)
Frequency of fish consumption ^7^	2.29 (0.85)	0.033 (0.974)	0.025 (0.744)	0.071 (0.362)
Frequency of fried food consumption ^8^	3.56 (0.92)	−2.853 (0.005)	0.165 (0.031)	−0.021 (0.784)
Frequency of sweets consumption ^8^	2.92 (1.08)	0.475 (0.636)	0.168 (0.028)	0.018 (0.813)
Importance of physical activity in life ^9^	4.42 (1.23)	4.296 (<0.001)	0.064 (0.409)	0.071 (0.361)
Frequency of physical activity ^10^	4.99 (1.02)	4.506 (<0.001)	0.180 (0.019)	0.132 (0.087)
Number of hours of physical activity per week ^11^	2.13 (0.92)	6.357 (<0.001)	0.164 (0.033)	0.206 (0.007)
Number of minutes of physical activity per day that increases heart rate and breathing	31.35 (22.84)	0.871 (0.385)	0.179 (0.021)	0.064 (0.415)
Organized physical activity ^12^	2.03 (0.72)	2.654 (0.011)	0.045 (0.588)	0.171 (0.039)
Unorganized physical activity ^13^	3.03 (0.99)	1.653 (0.100)	−0.015 (0.851)	0.001 (0.994)
Attention ^14^	2.98 (0.42)	−1.155 (0.252)	−0.060 (0.444)	−0.050 (0.530)
Difficulties sleeping due to worry ^15^	3.06 (0.95)	0.573 (0.568)	−0.070 (0.367)	−0.086 (0.271)
Feeling of constant pressure ^15^	2.90 (0.89)	0.163 (0.870)	−0.044 (0.573)	0.029 (0.712)
Feeling of losing self-confidence ^15^	3.34 (0.86)	0.853 (0.395)	−0.088 (0.261)	−0.116 (0.140)
Satisfaction with life ^14^	2.32 (0.67)	1.235 (0.219)	0.091 (0.240)	−0.069 (0.377)
Reason for changing lifestyle to improve physical well-being ^9^	4.88 (1.27)	−0.699 (0.486)	0.056 (0.464)	0.048 (0.535)
Reason for changing lifestyle to improve mental well-being ^9^	4.94 (1.25)	−3.200 (0.002)	−0.104 (0.175)	−0.088 (0.256)
Reason for lifestyle change for better health ^9^	4.73 (1.48)	−2.339 (0.022)	0.014 (0.858)	−0.081 (0.296)
Number of alcoholic beverages per week	3.67 (8.71)	2.415 (0.019)	−0.068 (0.376)	0.052 (0.500)
Smoking ^16^	3.72 (1.48)	0.403 (0.688)	0.009 (0.909)	0.046 (0.563)

Legend: ^1^ Mean value (M) and standard deviation (SD). ^2^ Coefficient and *p*-value of the statistical significance for *t*-test for independent samples. ^3^ Spearman’s *rho* (*ρ*) correlation coefficient and *p*-value of the statistical significance. ^4^ Body mass index. ^5^ Measured on a 6-point scale: 1 = least good to 6 = very good. ^6^ Measured on a 6-point scale: 1 = none to 6 = very good. ^7^ Measured on a 5-point scale: 1 = rarely or never to 5 = six or more times a week. ^8^ Measured on a 5-point scale: 1 = six or more times a week to 5 = rarely or never. ^9^ Measured on a 6-point scale: 1 = not important to 6 = very important. ^10^ Measured on a 7-point scale: 1 = never to 7 = every day. ^11^ Measured on a 4-point scale: 1 = zero or less than 3 h to 4 = more than 10 h. ^12^ Composite variable from six questions on different forms of organized exercise, which were assessed on a 6-point scale: 1 = very rarely or not at all to 6 = very often. ^13^ Composite variable from four questions on different forms of unorganized exercise, which were assessed on a 6-point scale: 1 = very rarely or not at all to 6 = very often. ^14^ Measured on a 4-point scale: 1 = much worse than usual to 4 = much better than usual. ^15^ Measured on a 4-point scale: 1 = much more than usual to 4 = much less than usual. ^16^ Measured on a 5-point scale: 1 = every day to 5 = never.

**Table 3:** Socio-demographic characteristics and naming of student group members according to their health-related behavior patterns.

Cluster Group	N (%)	Gender		Age	BMI ^2^
		Female %	Male %	M (SD) ^1^	M (SD)
CLU1 ^3^ “Caring for a healthy lifestyle”	69				
	(52.7)	42.4	84.4	21.97 (2.20)	22.49 (2.65)
CLU2 ^4^ “Physically inactive with poor mental well-being”	62				
	(47.3)	57.6	15.6	21.06 (1.67)	22.69 (4.71)
Total	100.0	100.0	100.0	100.0	100.0
*χ*^2^ (*p*) ^5^/*t* (*p*) ^6^		17.072		2.637	−0.302
		(<0.001)		(0.009)	(0.763)

Legend: ^1^ Mean value (M) and standard deviation (SD). ^2^ Body mass index. ^3^ CLU1—the first group of students, obtained using Ward’s hierarchical clustering method. ^4^ CLU2—the second group of students, obtained using Ward’s hierarchical clustering method. ^5^ Coefficient and *p*-value of the statistical significance for *t*-test for independent samples. ^6^ Coefficient and *p*-value of the statistical significance for chi-square test value.

## Data Availability

The dataset related to this manuscript can be made available upon reasonable request.
